# Quality of Colon Cancer Care in Patients Undergoing Emergency Surgery

**DOI:** 10.3390/curroncol28030192

**Published:** 2021-06-03

**Authors:** Keegan Guidolin, Rebecca Withers, Farhana Shariff, Shady Ashamalla, Ashlie Nadler

**Affiliations:** 1 Department of Surgery, University of Toronto, Toronto, ON M5T 1P5, Canada; keegan.guidolin@mail.utoronto.ca (K.G.); farhana.shariff@gmail.com (F.S.); shady.ashamalla@sunnybrook.ca (S.A.); 2 Department of Surgery, McMaster University, Hamilton, ON L8S 4K1, Canada; r.k.withers@gmail.com; 3 Sunnybrook Health Sciences Centre, Toronto, ON M4N 3M5, Canada

**Keywords:** colon cancer, emergency surgery, acute care surgery, quality of care, quality of cancer care

## Abstract

Thirty percent of colon cancer diagnoses occur following emergency presentations, often with bowel obstruction or perforation requiring urgent surgery. We sought to compare cancer care quality between patients receiving emergency versus elective surgery. We conducted an institutional retrospective matched (46 elective:23 emergency; *n* = 69) case control study. Patients who underwent a colon cancer resection from January 2017 to February 2019 were matched by age, sex, and cancer stage. Data were collected through the National Surgical Quality Improvement Program and chart review. Process outcomes of interest included receipt of cross-sectional imaging, CEA testing, pre-operative cancer diagnosis, pre-operative colonoscopy, margin status, nodal yield, pathology reporting, and oncology referral. No differences were found between elective and emergency groups with respect to demographics, margin status, nodal yield, oncology referral times/rates, or time to pathology reporting. Patients undergoing emergency surgery were less likely to have CEA levels, CT staging, and colonoscopy (*p* = 0.004, *p* = 0.017, *p* < 0.001). Emergency cases were less likely to be approached laparoscopically (*p* = 0.03), and patients had a longer length of stay (*p* < 0.001) and 30-day readmission rate (*p* = 0.01). Patients undergoing emergency surgery receive high quality resections and timely post-operative referrals but receive inferior peri-operative workup. The adoption of a hybrid acute care surgery model including short-interval follow-up with a surgical oncologist or colorectal surgeon may improve the quality of care that patients with colon cancer receive after acute presentations. Surgeons treating patients with colon cancer emergently can improve their care quality by ensuring that appropriate and timely disease evaluation is completed.

## 1. Introduction

Colon cancer is the fourth most commonly diagnosed malignancy and the second leading cause of cancer related death worldwide [1]. Approximately 30% of colorectal cancer cases are diagnosed following emergency presentations, typically due to obstructing tumours or bowel perforation [2,3,4]. Many studies have demonstrated that patients presenting emergently have greater disease burden and poorer outcomes compared to those managed in an elective setting. Emergency presentation itself has been identified as a poor prognostic factor, independent of disease stage [5].

The management of colon cancer, at its core, consists of surgical resection (for most patients), with the addition of adjuvant therapy for patients with nodal metastasis or high-risk tumour features [6]. High quality cancer care goes beyond this, and includes a host of additional components, including the pre-operative identification and histologic diagnosis of colon cancer, pre-operative staging computed tomography (CT) scanning, carcinoembryonic antigen (CEA) testing, pre-operative colonoscopy, pre-operative medical optimization, oncologic surgical resection including appropriate pathology requests, post-operative in-hospital management, short-interval outpatient follow-up, and timely referral to medical oncology (where indicated). Despite these metrics being both relevant and part of quality practice for most providers, they are infrequently captured in studies examining colon cancer care with research typically focusing on a narrow set of patient outcomes as study endpoints [7]. The exception to this is a group of studies investigating the rate of receipt of, and time to initiation of adjuvant chemotherapy; however, even these studies report conflicting results [8,9,10,11].

As the Acute Care Surgery (ACS) service model gains popularity across North America, more patients with advanced colon cancer are being managed outside of the usual efficient and comprehensive oncology workflow. We sought to evaluate process-based metrics not typically assessed in outcome-based studies, specifically looking at quality indicators for the population of patients who receive emergency surgery for their colon cancer as compared to surgical patients managed electively.

## 2. Materials and Methods

### 2.1. Study Setting and Design

**Figure 1:**
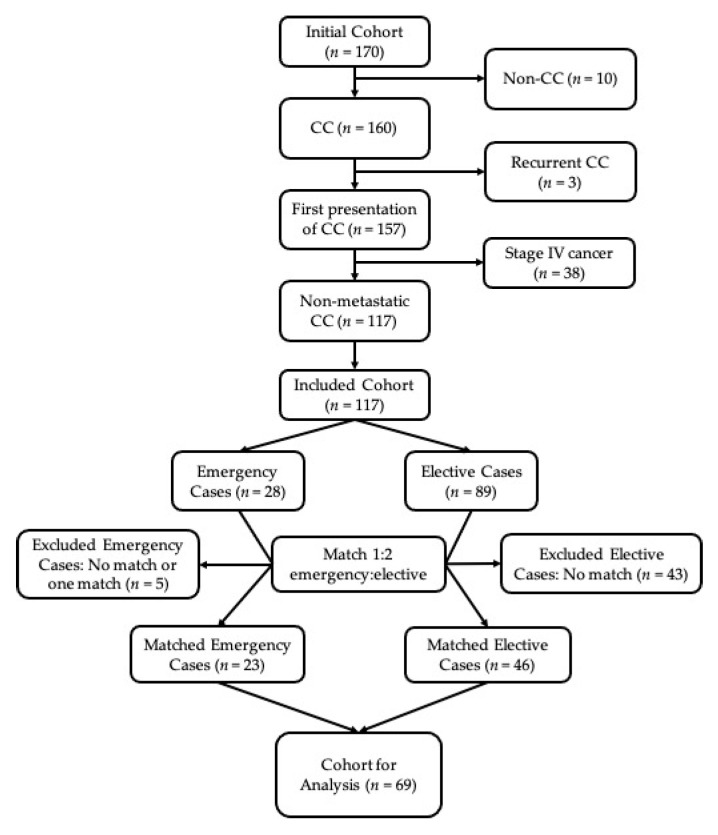
Cohort build. CC = colon cancer.

We conducted a matched case-control study on patients who underwent a colon cancer operation from 1 January 2017 to 28 February 2019 at an academic tertiary care center. This center manages elective patients through its cancer center, but patients presenting acutely through the emergency department are managed by the ACS service. Data were collected through the National Surgical Quality Improvement Program (NSQIP) and supplemented with additional information obtained from directed chart review. Exclusion criteria applied to the initial cohort (*n* = 170) were: non-resection/diversion procedures, rectal cancer, recurrent cancers, known stage IV disease, and non-adenocarcinoma disease. After exclusion criteria were applied, the initial cohort was divided into two groups: emergency (EM) cases (booked for surgery as an emergency case once the patient is admitted to hospital), and elective (EL) cases (underwent a planned operation in the elective operating schedule) based on procedure booking priority and confirmed by chart review. Each EM case was matched to two EL cases with respect to age (within 10 years), sex, and overall cancer stage. Patients who could not be matched 1:2 were excluded (5 EM cases and 43 EL cases). The final cohort used for analysis compared 23 EM cases to 46 EL cases (*n* = 69). Figure 1 illustrates the matched cohort build.

### 2.2. Outcomes and Baseline Variables

Patient baseline variables were compared to ensure that no significant differences existed between groups. Quality metrics of interest were selected to represent three aspects of colon cancer care: (i) peri-operative care, (ii) surgical care, and (iii) continuity of care. Specifically, outcomes of interest included (i) CT staging (i.e., CT chest, abdomen, and pelvis) and CEA within 30 days of surgery, pre-operative colonoscopy, pre-operative cancer diagnosis, and pre-operative pathological diagnosis; (ii) nodal yield, margin status, time from surgery to pathology report, length of stay, and 30-day readmission, and; (iii) time from surgery to medical oncology referral, and time to follow-up appointment. 

### 2.3. Statistical Analysis

Univariate analysis was conducted using Pearson chi-square (for nominal variables), and Mann Whitney U (for continuous variables) testing in SPSS.

### 2.4. Ethics Approval

The study was approved by the Sunnybrook Health Sciences Centre Research Ethics Board (REB #080-2019).

## 3. Results

### 3.1. Baseline Variables

Baseline variables for our cohort are shown in Table 1. More patients undergoing EL were ASA class III (74% vs. 39%, *p* = 0.01), while more EM patients were ASA class IV (57% vs. 17%, *p* = 0.01). No differences existed in patient age, gender, tumour location, or disease stage. There were also no differences in pre-operative cancer diagnosis (clinical or pathologic) and pre-operative pathology status. EM cases were most commonly conducted for obstruction (*n* = 15, 65.2%), followed by bleeding (*n* = 4, 17.4%), and perforation (*n* = 1, 4.4%); in three cases, the indication for emergency (rather than elective) surgery was not specified.

Those undergoing EL were more likely to have had a surgeon with fellowship training in either surgical oncology or colorectal surgery compared to those undergoing EM (100% vs. 91%, *p* = 0.042). More patients undergoing EL had their procedures done using a laparoscopic approach (85% vs. 39%, *p* = 0.001), while EM patients had a higher rate of open or laparoscopic converted to open approaches (48% vs. 11%, *p* = 0.001). This trend was reflected in the procedure performed, with more open right hemicolectomies performed in EM patients (30% vs. 9%, *p* = 0.046). 

### 3.2. Outcome Comparison

This study found no differences between EM and EL surgery with respect to margin status, nodal yield (a surrogate of oncologic quality of the operation), receipt of appropriate medical oncology referral, or time from operation to medical oncology referral (continuity of care). The time from operation to pathology report approached significance with EM cases waiting longer for pathology reporting than EL cases (16 days vs. 13.5 days, *p* = 0.058). With respect to peri-operative metrics, EM patients were less likely to receive CT staging (including CT chest, abdomen, and pelvis) within 30 days of their operation (87% vs. 100%, *p* = 0.012), and CEA baseline levels measured within 30 days of their operation (70% vs. 97%, *p* = 0.02). Those undergoing EL were more likely to have colonoscopies completed pre-operatively (95.7% vs. 69.6%, *p* < 0.001) than those undergoing EM. These results are shown in Table 2.

## 4. Discussion

**Figure 2:**
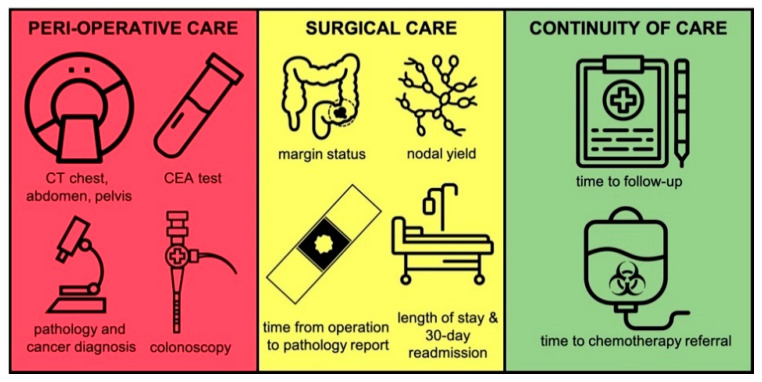
The three moments of cancer care.

Our study sought to assess process-based cancer care quality metrics that are rarely assessed in outcome-based studies but are nonetheless essential to providing excellent care. The importance of quality metrics in emergency surgery outside of the traditionally studied perioperative outcomes has been emphasized in recent literature: a Delphi expert consensus identified appropriate guideline directed follow-up and surveillance (per National Comprehensive Cancer Network guidelines) for patients undergoing emergency oncology operations as a metric of quality of care [12]. We divided the cancer care workflow into three “moments” and selected processes we perceived to represent the quality of care provided at each moment. The moments we identified were: (i) peri-operative care, (ii) surgical quality, and (iii) continuity of care (Figure 2). For the peri-operative care moment, we identified complete CT staging (chest, abdomen, and pelvis), CEA levels within 30-days of the operation, pre-operative cancer diagnosis, pre-operative pathology status, and pre-operative colonoscopy as factors representing excellent care. For the surgical quality moment, we identified margin status, nodal yield, and time from operation to pathology report, as factors representing excellent care. At our institution, surgical specimens from elective cancer cases are automatically flagged for urgent pathological analysis to facilitate adjuvant therapy planning, if needed. We hypothesized that EM cases would have a greater delay from surgery to pathology result because this systematic approach is lost and the responsibility to mark a specimen as “urgent” falls to the individual surgeon. This delay would also, theoretically have had implications for timely referral for, and receipt of adjuvant therapy.

The continuity of care management moment includes medical oncology referral (as appropriate based on pathology results), time from operation to medical oncology referral, and time to surgical follow-up appointment as factors representing excellent care. 

Our findings suggest the maintenance of excellent cancer care, even in the setting of emergency surgery with respect to most aspects of the care pathway. The quality of surgical care (represented by margin status and nodal yield) was no different between groups. This finding is supported by existing literature which suggests that emergency surgery is associated with superior nodal yield compared with elective surgery [8,13,14], although others provide contradictory results [11,15]. While the length of stay is significantly greater in EM cases, this is an expected (and well published) finding and likely serves as a marker of the overall pre-operative health of this patient population [16,17]. This interpretation is supported specifically by our findings that ASA class was higher in this group. Finally, we had expected to find a significantly longer interval from operation to pathology reporting based on subjective experience; however, according to our analysis, there was no significant difference. Though this association approached significance, the median difference between the groups was 2.5 days—a difference that is unlikely to be clinically significant. This effect may be due to specimens being marked for “urgent” pathological analysis despite cases being performed in the emergency setting because in our center, many of the operating ACS surgeons are trained in colorectal surgery or surgical oncology. This does not appear to have been previously studied and so comparison to existing literature is not possible, though we consider this to be an interesting metric for future study. 

Cancer care quality also seems to be preserved at the continuity of care management moment, with both groups receiving appropriate and timely referrals to medical oncology. Current literature in this domain is limited, with one study at a tertiary care center corroborating our findings [8]; however, a French study of 24 regional hospitals found that emergency surgery was independently associated with longer time to receipt of adjuvant chemotherapy [18]. Several studies have also found an association between emergency surgery and non-receipt of adjuvant chemotherapy [10,11]. This discrepancy is likely multifactorial, but contributing factors may include national guideline differences, practice setting, surgeon training, or other unmeasured confounders.

According to our analysis, the greatest disparity between the urgency levels of patients occurs during the “peri-operative care” moment. While almost all EL patients received timely staging CT scans and CEA level measurements, only 87% of EM patients received staging CT scans, and only 70% had CEA levels measured. Similarly, almost all EL patients had a pre-operative colonoscopy completed, while far fewer EM patients had the procedure completed (69.6%, *p* < 0.001). These investigations provide valuable information for both the short- and long-term management of colon cancer. In the elective setting these investigations are typically completed prior to surgery. Recognizing that the nature of emergency presentations does not always lend itself to such management, we allowed for a delay of up to 30 days following the operation to complete this work-up. Despite this allowance, a significant proportion of patients undergoing EM failed to receive what we would consider to be “excellent” peri-operative care. This highlights an area for improvement in the delivery of quality colon cancer care to patients presenting emergently, as these particular quality of care metrics do not appear to have been examined in the literature.

Our study is limited by its retrospective, single-centre nature, and its relatively small sample size. Unfortunately, the granular data points that we sought to analyze are not routinely collected in any population-based dataset, and so the only way to collect this data is by institutional chart review. 

Patients who undergo emergency colon cancer resection comprise a unique group of patients with specialized needs that are not always met on a typical ACS service. In order to better meet the needs of such patients, we propose a hybrid ACS system wherein these patients would have their surgical procedure performed by the ACS surgeon on call if needed but would be referred to a surgical oncologist or colorectal surgeon upon admission for peri-operative input, short-interval follow-up, and post-operative management in general. In our center, patients are referred to our ACS clinic for post-operative follow-up, which is staffed by a surgical oncologist, helping to ensure that appropriate investigations, referrals, and surveillance are completed. Moreover, immediate inpatient referral may facilitate coordination of appropriate investigations and referrals before the patient is discharged from their index admission. 

Future work in this area should examine these trends in a wide variety of practice settings (e.g., academic, urban community, rural), to determine how practice setting might be associated with these cancer care quality metrics. In addition, more detailed data would optimize future studies on the time from surgery to the actual initiation of adjuvant chemotherapy; this was not available to us because many patients were referred to community hospitals closer to their home for chemotherapy and the records are not available to our center. Finally, future research could study such process benchmarks in the management of colon cancer using other techniques, for instance for those undergoing diversion without resection, to identify whether the strengths and weaknesses found here are maintained across the spectrum of colon cancer.

## 5. Conclusions

We identified that patients undergoing emergency colon cancer surgery receive surgical care and continuity of care that is comparable in quality to those who undergo elective resection; however, patients undergoing emergency surgery are less likely to receive a complete cancer work-up peri-operatively, including complete CT staging (chest, abdomen, pelvis), colonoscopy, and CEA levels. We propose a hybrid ACS model to address the unique needs of colon cancer patients.

## Figures and Tables

**Table 1:** Baseline variables.

Variable	Emergency Cases (*n* = 23)	Elective Cases (*n* = 46)	*p*-Value
Age (years), median (IQR)	73 (64.4–79.7)	73.7 (63.3–81.7)	0.843
Male sex (%)	13 (56.5%)	26 (56.5%)	1.00
ASA Class (%)			0.01
I	0 (0%)	1 (2.2%)	
II	1 (4.4%)	3 (6.5%)	
III	9 (39.1%)	34 (73.9%)	
IV	13 (56.5%)	8 (17.4%)	
V	0 (0%)	0 (0%)	
BMI, median (IQR)	31.9 (27.4–34.7)	27.9 (24.1–30.9)	0.412
Subspecialized Surgeon ^a^ (%)	21 (91.3%)	46 (100%)	0.042
Type of Operation			0.046
Open right hemicolectomy (%)	7 (30.4%)	4 (8.7%)	
Laparoscopic right hemicolectomy (%)	9 (39.1%)	23 (50.0%)	
Segmental colectomy (%)	2 (8.7%)	0 (0%)	
Laparoscopic segmental colectomy (%)	3 (13.0%)	9 (19.6%)	
Laparoscopic low anterior resection (%)	1 (4.4%)	8 (17.4%)	
Open low anterior resection (%)	0 (0%)	1 (2.2%)	
Laparoscopic low anterior resection + diversion (%)	1 (4.4%)	0 (0%)	
Laparoscopic Hartmann’s procedure (%)	0 (0%)	1 (2.2%)	
Approach			0.001
Open (%)	9 (39.1%)	3 (6.5%)	
Laparoscopic (%)	9 (39.1%)	39 (84.8%)	
Converted (%)	2 (8.7%)	2 (4.4%)	
Laparoscopic Assisted (%)	3 (13.0%)	2 (4.4%)	
Tumour Location			0.468
Right (%)	15 (65.2%)	27 (58.7%)	
Transverse (%)	3 (13.0%)	4 (8.7%)	
Left (%)	2 (8.7%)	2 (4.4%)	
Sigmoid (%)	3 (13.0%)	13 (28.3%)	
Cancer diagnosed pre-operatively			0.108
Yes	20 (87%)	45 (97.8%)	
No	1 (4.4%)	1 (2.2%)	
On differential but unconfirmed	2 (8.7%)	0 (0%)	
Pre-operative pathology status			0.299
Negative	2 (8.7%)	2 (4.4%)	
Positive	14 (60.9%)	40 (87.0%)	
Pre-operative colonoscopy			<0.001
Yes	16 (69.6%)	44 (95.7%)	
No	7 (30.4%)	0 (0%)	
Attempted but uncompleted	0 (0%)	2 (4.4%)	
Pathologic T Stage			0.092
1 (%)	0 (0%)	1 (2.2%)	
2 (%)	1 (4.4%)	6 (13.0%)	
3 (%)	15 (65.2%)	35 (76.1%)	
4 (%)	7 (30.4%)	4 (8.7%)	
Pathologic N Stage			0.255
0 (%)	11 (47.8%)	22 (47.8%)	
1 (%)	4 (17.4%)	15 (32.6%)	
2 (%)	8 (34.8%)	9 (19.6%)	
Pathologic M Stage			1.000
0 (%)	23 (100%)	46 (100%)	
1 (%)	0 (0%)	0 (0%)	

^a^ Surgeons with fellowship training in either surgical oncology or colorectal surgery.

**Table 2:** Comparison of outcomes between patients undergoing emergency versus elective colon cancer surgery.

Variable	Emergency Cases (*n* = 23)	Elective Cases (*n* = 46)	Odds Ratio (95% CI)	*p*-Value
CT staging within 30 days (%)	20 (87%)	46 (100%)	N/A	0.012
CEA within 30 days (%)	16 (70%)	44 (97%)	0.104 (0.020–0.553)	0.020
Longitudinal margin				1.000
Gross negative	23 (100%)	46 (100%)	N/A	
Microscopic negative	23 (100%)	46 (100%)	N/A	
Circumferential margin				
Gross negative	23 (100%)	46 (100%)	N/A	1.000
Microscopic negative	20 (87%)	45 (97.8%)	N/A	0.069
Nodal yield, median (IQR)	28 (19–33.5)	27 (20–37)	N/A	0.819
Time from operation to pathology report, median days (IQR)	16 (13.5–19.5)	13.5 (10.25–18.75)	N/A	0.058
Length of stay, days, median (IQR)	9 (6–13)	2.5 (1–5)	N/A	<0.001
30-day readmission (%)	6 (26%)	2 (4%)	3.706 (0.928–14.803)	0.053
Medical oncology referral ^a^	16 (70%)	39 (85%)	0.737 (0.236–2.303)	0.599
Time from operation to medical oncology referral, days, median (IQR)	30 (18–33)	27 (21.5–34)	N/A	0.346

^a^ High risk stage II or any stage III.

## Data Availability

The data presented in this study are available on request from the corresponding author. The data are not publicly available due to our institutional privacy bylaws.
